# Supplementary material for: Complex‐centric proteome profiling by SEC‐SWATH‐MS
Source: Mol Syst Biol. 2019 Jan 14;15(1):e8438. doi: 10.15252/msb.20188438 (PMC6346213; doi:10.15252/msb.20188438)
Supplement: Supplementary file 8 — Dataset EV7 [file MSB-15-e8438-s008.zip › feature_plots_string/P28331.pdf]

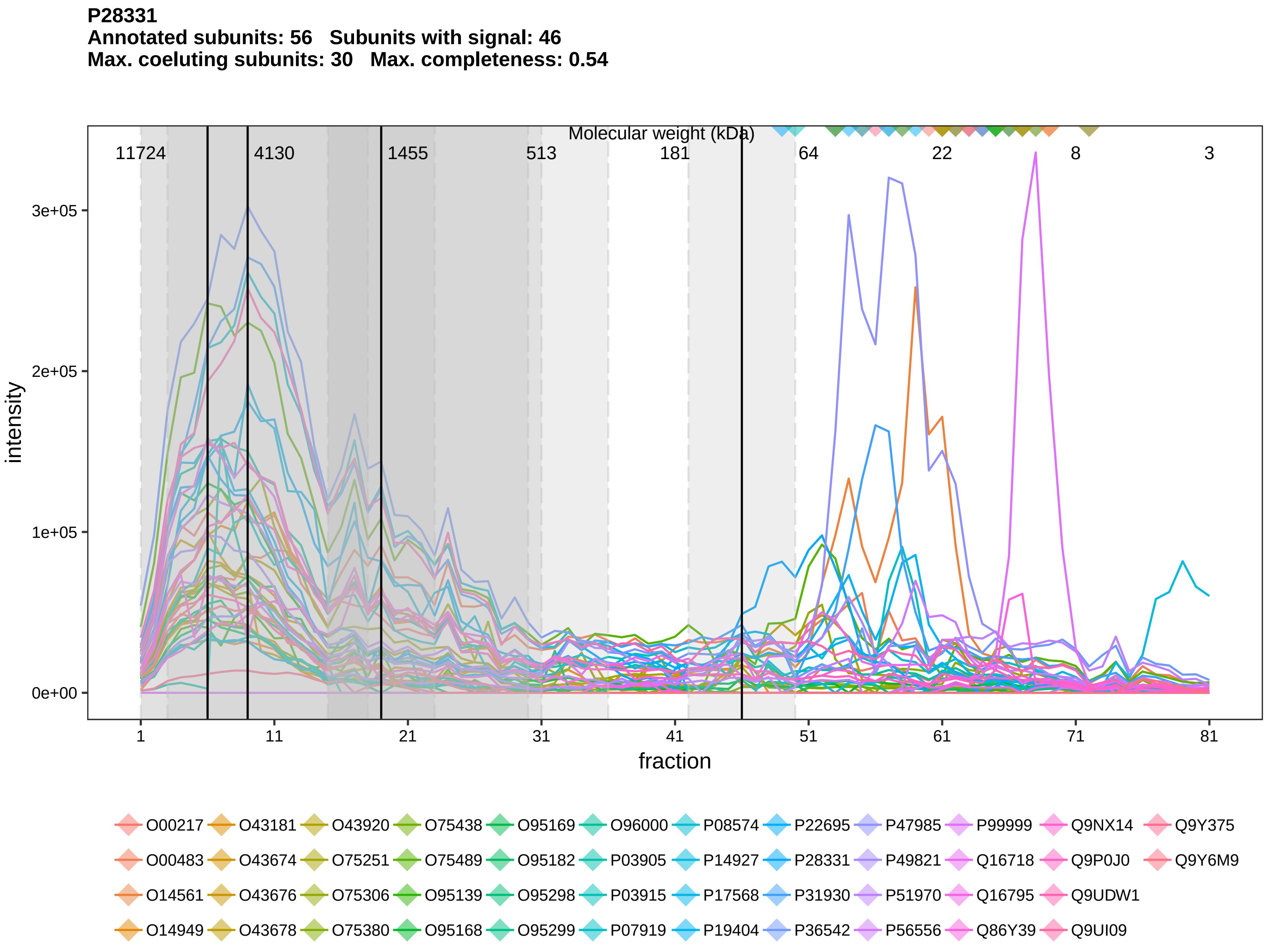
P28331
Annotated subunits: 56 Subunits with signal: 46
Max. coeluting subunits: 30 Max. completeness: 0.54
Molecular weight (kDa)
11724
4130
1455
513
181
64
22
8
3
intensity
3e+05
2e+05
1e+05
0e+00
fraction
1
11
21
31
41
51
61
71
81
O00217
O00483
O14561
O14949
O43181
O43674
O43676
O43678
O43920
O75251
O75306
O75380
O75438
O75489
O95139
O95168
O95169
O95182
O95298
O95299
O96000
P03905
P03915
P07919
P08574
P14927
P17568
P19404
P22695
P28331
P31930
P36542
P47985
P49821
P51970
P56556
P99999
Q16718
Q16795
Q86Y39
Q9NX14
Q9P0J0
Q9UDW1
Q9UI09
Q9Y375
Q9Y6M9
